# Heart rate-related physiological changes induced by classical music-elicited emotions do not underlie alterations in healthy adults’ ankle joint target-matching strategy

**DOI:** 10.1038/s41598-024-67467-y

**Published:** 2024-07-17

**Authors:** Keqing Yuan, Takeshi Okuyama, Tibor Hortobágyi, Ryoichi Nagatomi, János Négyesi

**Affiliations:** 1 Department of Medicine and Science in Sports and Exercise, Tohoku University Graduate School of Medicine, Sendai, Japan; 2 Department of Robotics, Tohoku University Graduate School of Engineering, Sendai, Japan; 3 Department of Kinesiology, Hungarian University of Sports Science, Pf. 69., Budapest, 1525 Hungary; 4 Department of Human Movement Sciences, Center for Human Movement Sciences, University Medical Center Groningen, University of Groningen, Groningen, The Netherlands; 5 Institute of Sport Sciences and Physical Education, University of Pécs, Pecs, Hungary; 6 Department of Biomedical Engineering for Health Maintenance and Promotion, Graduate School of Biomedical Engineering, Tohoku University, Sendai, Miyagi Japan; 7 Neurocognitive Research Center, Nyírő Gyula National Institute of Psychiatry, and Addictology, Budapest, Hungary; 8 CRU Hungary Kft., Budapest, Hungary

**Keywords:** Auditory perception, Ankle joint, Emotions, HRV, Proprioception, Auditory system, Emotion, Motor control, Sensorimotor processing

## Abstract

Emotions have the potential to modulate human voluntary movement by modifying muscle afferent discharge which in turn may affect kinesthetic acuity. We examined if heart rate (HR)-related physiological changes induced by music-elicited emotions would underlie alterations in healthy young adults’ ankle joint target-matching strategy quantified by joint position sense (JPS). Participants (n = 40, 19 females, age = 25.9 ± 2.9 years) performed ipsilateral-, and contralateral ankle target-matching tasks with their dominant and non-dominant foot using a custom-made foot platform while listening to classical music pieces deemed to evoke happy, sad, or neutral emotions (each n = 10). Participants in the 4th group received no music during the task. Absolute (ABS), constant (CONST), and variable (VAR) target-matching errors and HR-related data were analyzed. Participants performed the contralateral target-matching task with smaller JPS errors when listening to sad vs. happy music (ABS: p < 0.001, d = 1.6; VAR: p = 0.010, d = 1.2) or neutral (ABS: p < 0.001, d = 1.6; VAR: p < 0.001, d = 1.4) music. The ABS (d = 0.8) and VAR (d = 0.3) JPS errors were lower when participants performed the task with their dominant vs. non-dominant foot. JPS errors were also smaller during the ipsilateral target-matching task when participants (1) listened to sad vs. neutral (ABS: p = 0.007, d = 1.2) music, and (2) performed the target-matching with their dominant vs. non-dominant foot (p < 0.001, d = 0.4). Although emotions also induced changes in some HR-related data during the matching conditions, i.e., participants who listened to happy music had lower HR-related values when matching with their non-dominant vs. dominant foot, these changes did not correlate with JPS errors (all p > 0.05). Overall, our results suggest that music-induced emotions have the potential to affect target-matching strategy and HR-related metrics but the changes in HR-metrics do not underlie the alteration of ankle joint target-matching strategy in response to classical music-elicited emotions.

## Introduction

Emotions affect the control of voluntary movement and motor performance. Emotions exert an effect by modulating readiness to move^1^. The fight or flight response is an example for this emotional modulation. In such responses, proprioception is a fundamental element of voluntary motor control because active movements require the brain to process afferent signals from proprioceptors. Muscle spindles, located in the muscle belly, provide information about the length and rate of stretch^2^. Indeed, emotional context can strongly modulate muscle afferent firing so that sad emotions increase muscle spindles’ dynamic response^3^. Emotional states seem to prime voluntary movements and facilitate the selection of the subsequent behavior-appropriate reactions because emotion can modify the sensitivity of muscle afferent activity^4,5^, suggesting that emotions can modulate muscle feedback directly. Previous reserach^6^ has determined that emotional states modify kinesthetic acuity, i.e., feelings of sadness increase the ability to detect movements and reliably report their direction.

A broad spectrum of methods has been developed to measure features of proprioceptive accuracy^7^. Joint position sense (JPS), the perceived sense of joint position^8^, is a valid method to assess proprioceptive acuity. Ankle joint proprioceptive acuity is fundamental for maintaining standing and walking balance, adjusting posture while ambulating, and reducing the risk of falls^9,10^. For example, smaller ankle proprioceptive errors are associated with higher motor performance, measured by an active movement extent discrimination test^11^. Ankle JPS tasks are, therefore, widely used in both healthy^12,13^ and clinical^14,15^ populations. Target-matching tasks can be done contralaterally and ipsilaterally. It is recommended to execute the measurements on each side in both conditions because the contralateral tasks involve interhemispheric transfer of information, while the ipsilateral tasks are memory-based^16,17^. Another important difference between the two types of target-matching tasks is the magnitude of errors, i.e., participants performing contralateral vs. ipsilateral tasks tend to produce larger errors^7,18^.

Overall, emotions modify muscle afferent firing which in turn seems to affect kinesthetic acuity^4–6^. Nevertheless, there is a need to further clarify if one particular physiological outcome, that is known to be affected by emotions, underlies the effects of music-induced emotions on target-matching tasks in the dominant and non-dominant limbs. The purpose of this study is to examine if heart rate (HR)-related physiological changes induced by music-elicited emotions would underlie alterations in healthy young adults’ ankle joint target-matching strategy quantified by JPS. Evidence suggests that emotions bidirectionally affect heart rate variability (HRV): positive emotions increase while negative emotions decrease HRV^6,19,20^. Because emotions can modulate muscle afferent discharge^4,5^ and these afferents also control JPS^6^, it is conceivable that changes in HRV induced by sad or happy music are related to ankle JPS error measured in healthy young individuals. This idea is also supported by the indirect connection between some proprioceptive afferents and the autonomic nervous system^21^, which is often characterized by HRV, the noninvasive indicator of its function^22^. Thus, we hypothesized that generating classical music-elicited emotions will affect HRV which in turn will affect ankle JPS errors. Specifically, we hypothesized that sad emotions will reduce JPS errors due to the increased muscle spindle dynamic response shown in a previous study^3,6^. We also hypothesized that participants who listen to classical music pieces that elicit happy emotions will perform the proprioceptive acuity task with larger errors due to the greater variability in muscle spindle dynamic firing^3^. Lastly, in line with previous studies^23,24^, we hypothesized that participants will perform the target-matching tasks more accurately with their non-dominant vs. dominant limb, regardless of their emotional state and the task (contralateral, ipsilateral) due to the right hemisphere dominance during proprioceptive position sense supported by neuroimaging data^17^. The present study fits under the current efforts to understand how emotions affect motor control. This is an important research question as external stochastic noise can improve motor performance^25^ and postural control^26^ and such favorable effects have been demonstrated in patients with movement^27^ or affective^28^ disorders.

## Materials and methods

### Participants

Sample size calculations (G*Power 3.1.7^29^) based on a previous study^3^ that aimed to determine the effects of emotions on descending motor drive, revealed that a total sample size of 39 would be appropriate to detect significant differences between the groups, assuming type I error of 0.05, power of 0.80, numerator degrees of freedom (df) of 9, number of 4 groups and effect size of 0.72 (from η_p_^2^ = 0.34). In the present parallel randomized single-blinded pilot study, we recruited 40 participants (age = 25.9 ± 2.9 years; height = 1.71 ± 0.07 m; mass = 63.5 ± 13 kg; 19 females) with no history of orthopedic or neurological disorders and were randomly assigned to one of the four groups. Participants in different groups listened to different classical musical pieces that were deemed to evoke (1) happy, (2) sad, or (3) neutral emotions. Participants in the fourth group received no music during the experiment. None of the participants had experience with the JPS tasks. After giving both verbal and written explanations of the experimental protocol, participants signed the informed consent document in accordance with the declaration of Helsinki. Local ethical permission was given by the Ethical Commission of Tohoku University (Approval No. 2022-1-140).

### Experimental procedures

**Figure 1:**
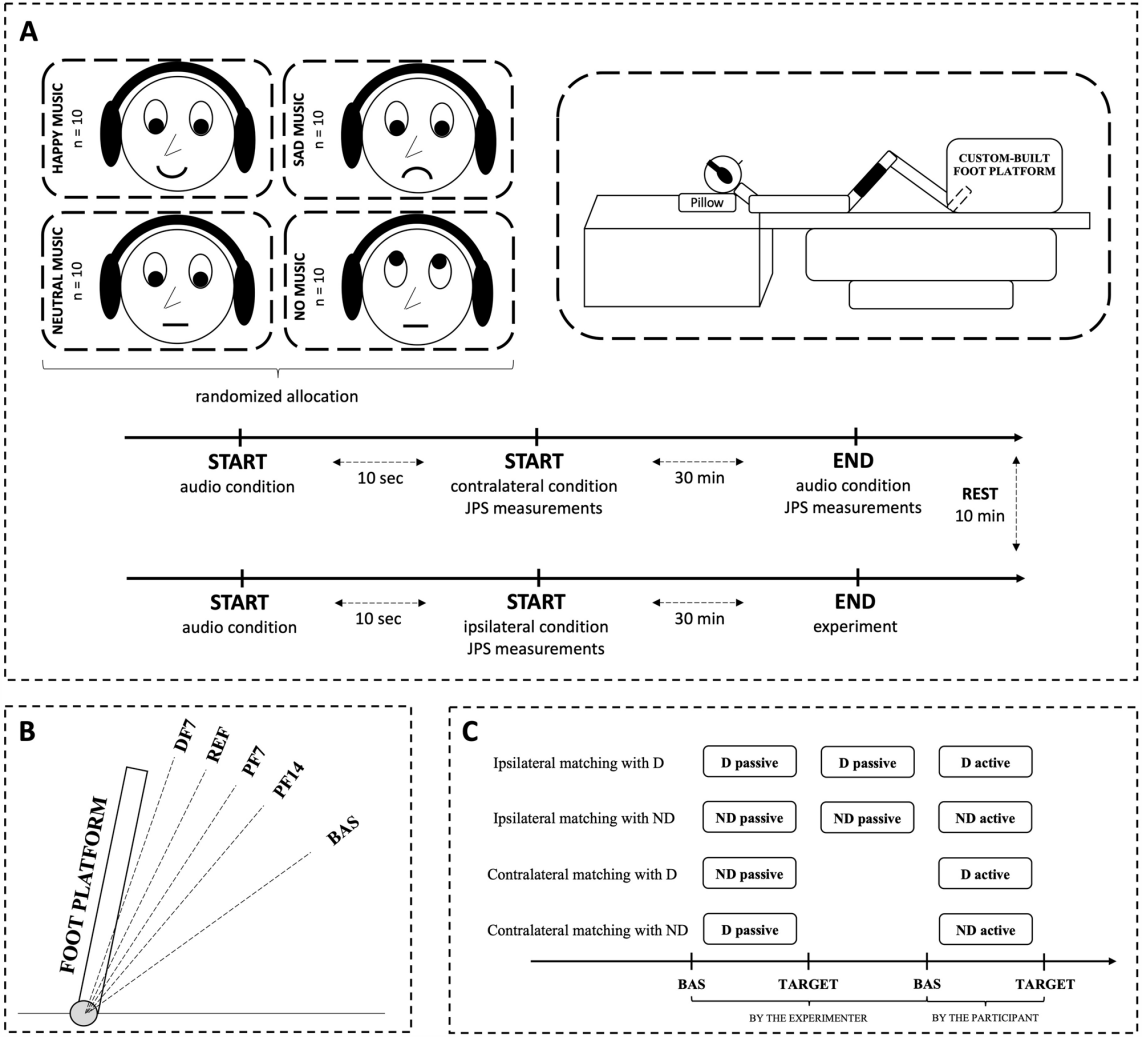
Experimental setup. (**A**) Participants performed 48–48 (4 targets × 4 trials × 3 blocks) ipsilateral-, and contralateral ankle JPS tasks using a custom-made adjustable foot platform. During the experiment, participants listened to a piece of classical music deemed to evoke happy, sad, or neutral emotions. The most effective music was selected before the experiment based on the participants’ subjective rating. Participants in the control group received no music during the experiment. (**B**) The initial position and the target angles of JPS tasks. The foot platforms could be locked at four predefined positions (target positions), each separated by 7° from the nearest positions: REF (90°), DF7 (7° dorsiflexion), PF7 (7° plantarflexion) and PF14 (14° plantarflexion). Each trial started with both feet from BAS. (**C**) During IL, the experimenter passively moved the participants’ R or L foot from BAS to one of the four target positions. After the experimenter repositioned the foot in the BAS position, participants had to match the previous position with the same foot and after holding it for a few seconds, return to the BAS position. During CL, the experimenter moved the foot to one of the four target positions, and then the participant had to reach the selected position with the contralateral foot. When the participant reached the position, both feet were repositioned to the BAS position. *BAS* baseline resting position, *D* dominant foot, *DF* dorsiflexion, *JPS* joint position sense, *ND* non-dominant foot, *PF* plantarflexion, *REF* reference position with the foot oriented at 90° concerning the legs.

Figure 1A shows the schematic illustration of the experimental design. Participants performed ipsilateral-, and contralateral ankle JPS tasks using a custom-made adjustable device allowing for independent movements of each foot in the sagittal plane. The platform consists of a foot fixation plate, two rotary encoders (E6C2-CWZ1X 2000P/R, OMRON Corporation), and an aluminum frame for mounting them. The encoder is connected to a data recording device (USB-6210, National Instruments Corp.) and software (LabVIEW, National Instruments Corp.) to identify the ankle joint angles during the experiment. Each acrylic plate on which the feet are placed is fixed to an aluminum frame, which is then fixed to the rotary axis of the encoder. The position of the acrylic plate can be adjusted so that the position of the ankle joint is aligned with the central axis of the rotary encoder. The axis is supported by a bearing designed to reduce the frictional resistance derived from the platform in the rotation of the ankle joints. The encoder is directly connected to the foot fixation plate without any gear; therefore, its resolution is the same as the resolution of the joint angle measurement (360/2000 = 0.18°). The left and right foot angle measurement mechanisms can rotate individually and are designed so that the rotational axes of both sides are aligned.

Before the experiment, participants listened to several pieces of classical music deemed to evoke happy, sad, or neutral emotions (up to 5 pieces of each type), and rated each of them on a visual analogue scale (VAS), ranging from sad (equating to 0, the saddest they could feel) to happy (equating to 10, the happiest they could feel). For each participant, the most effective music in triggering each type of emotion, for sad, neutral, and happy pieces were chosen to be played during the physiological recordings in each condition. The same set of signal-processed classical musical pieces was used then in a previous study^3^ (Supplementary Table 1).

Figure 1B shows the initial position and the target angles of JPS tasks. The platforms’ rotation angle was sampled at 100 Hz using incremental optical encoders. The experimental setup of the JPS measurements was done based on previous research^17^, which also examined JPS tasks with the ankle joint. Participants were positioned in the device with the foot oriented at 90° concerning the legs (REF). Therefore, the upward rotation of the platform with respect to this reference position required ankle dorsiflexion (DF) while the downward rotations required ankle plantarflexion (PF). The foot platforms could be locked at four predefined positions (target positions), each separated by 7° from the nearest positions: REF (90°), DF7 (7° dorsiflexion), PF7 (7° plantarflexion) and PF14 (14° plantarflexion). Each trial started with both feet from a baseline resting position (BAS). We tested 2 positions in the plantarflexion range because its range of motion is larger than the dorsiflexion’s^30^.

Participants performed the ipsilateral-, and contralateral matching tasks (Fig. 1C) with a single movement without corrections while approaching the target. Once the participants reached the target position, they had to maintain it for 3 s before moving their foot back to the BAS. During the ipsilateral matching task, the experimenter passively moved the participants’ dominant or non-dominant foot from the starting position to one of the four target positions. After the experimenter repositioned the foot in the BAS position, participants had to match the previous position with the same foot and after holding it for a few seconds, return to the BAS position. During the contralateral matching task, the experimenter moved the foot to one of the four target positions, and then the participant had to reach the selected position with the contralateral foot. When the participant reached the position, both feet were repositioned to the BAS position. After 5 trials of familiarization in each condition, participants performed 48–48 ipsilateral and contralateral matching trials, i.e., each target position was reached 4 times with 5 s of rest allowed between each trial in one block with both the dominant and non-dominant foot (4 targets × 4 trials × 3 blocks). To minimize learning effects, the order of all tasks (ipsilateral or contralateral) and target angles were randomized using random number generation in Microsoft Excel (version 16.83, Microsoft Corporation, Redmond, WA) (Supplementary Data 1–4). Participants kept their eyes closed during the experiment and received no feedback on their performance. In addition to the absolute target-matching errors (ABS), constant (CONST) and variable (VAR) errors were also evaluated to provide additional information on the integrity of the sensorimotor system by reflecting how accurately the target is represented in the nervous system^31,32^.

Target-matching errors were calculated in line with previous studies^33,34^:

Any deviation from the target position, discounting direction, was defined as the absolute position error:

1 $$ ABS \, = \, |{X_{participant}} - \, {X_{target}}| $$

For constant error, the difference between reproduced and actual target angle was used, considering the direction of the error:

2 $$ CONST \, = \, \left( {{X_{participant}} - \, {X_{target}}} \right) $$

The variable error was calculated as the overall standard deviation (SD) of constant error from 48 trials, irrespective of the target range:

3 $$\text{VAR }= {\sqrt{\sum (\text{X}{\text{participant}} -\text{E}{\text{constant}})}}^{2}$$

Recordings of heart rate (HR) and RR intervals were taken continuously during the JPS tasks with a Polar H10 sensor chest strap device (Polar Electro Oy, Kempele, Finland; sampling rate: 1000 Hz; app software: Elite HRV App, Version 5.5.1), which was reported to produce valid and accurate heart rate variability (HRV) data^35^. The Polar flow sync app was used to export the raw data, and the HRV analysis was done using the Kubios HRV Standard software (3.3.1). To provide insights into the autonomic nervous system's influence on cardiac regulation during JPS tasks, a widely used^36,37^ index, the ratio of low frequency to high frequency (LF/HF) of HRV was used. The normal value of LF/HF is 2.8 ± 2.6. If the LF/HF ratio value is higher, it denotes sympathetic nervous system (SNS) domination, and if the value is lower, it denotes parasympathetic nervous system (PNS) domination.

### Statistical analyses

Statistical analyses were performed using SPSS Statistics Package (version 28.0.1, SPSS Inc., Chicago, IL, USA). All data were checked for normal distribution by Shapiro–Wilk’s test and visual inspection of their histograms. Log transformation was used for variables that were not normally distributed. The analyses were done on the transformed data but all variables are reported in their original, non-transformed, form as mean ± standard deviation (SD). A series of mixed analysis of variance (ANOVA) was applied to assess the effects of emotions (grouping variable: happy, sad, neutral, no music) on the laterality (independent variable: leg [dominant and non-dominant foot]) of JPS (ABS, CONST, VAR) and the corresponding HR-related (mean, min, max, median, HRV) variables. In case of a significant main effect, planned post-hoc tests with Bonferroni correction for multiple comparisons were performed. The Greenhouse–Geisser correction was used when data violated the assumption of sphericity. Additionally, effect sizes of repetition factors were expressed using partial eta squared (η_p_^2^)^38^. Complementary post-hoc analyses (independent-samples t-tests for Group main effect, paired-samples t-tests for Leg main effect) were used when indicated. Cohen’s effect size (*d*) was also computed as appropriate. In order to determine if HR-related physiologic changes were associated with JPS errors in each condition, Pearson’s correlation was computed. Statistical significance was set at p < 0.05.

### Ethics statement

Local ethical permission was given by the Ethical Commission of Tohoku University (Approval No. 2022-1-140) and all experiments were conducted according to the latest version of the declaration of Helsinki. After giving both verbal and written explanations of the experimental protocol, participants signed the informed consent document.

## Results

### Contralateral matching task

**Figure 2:**
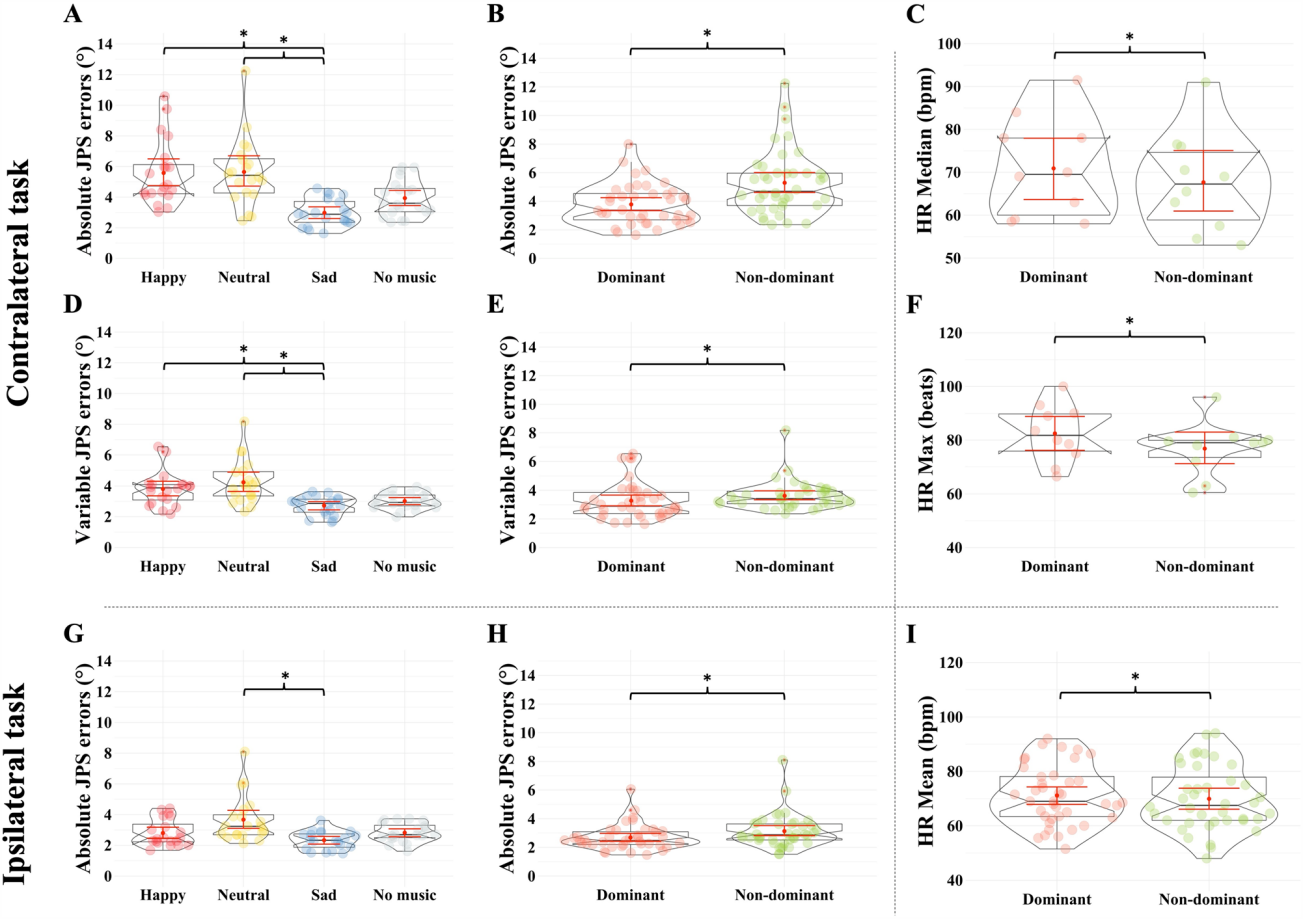
The effects of classical music-elicited emotions on target-matching errors and heart rate-related data during contralateral and ipsilateral tasks with both feet. (**A**) Participants performed the contralateral target-matching task with smaller ABS JPS errors when listening to sad music as compared to happy or neutral music. (**B**) Participants performed the contralateral target-matching tasks with smaller ABS JPS errors when matching with their dominant vs. non-dominant feet. (**C**) Participants who listened to happy music during the contralateral target-matching tasks had lower median heart rate when matching with their non-dominant vs. dominant feet. (**D**) Participants performed the contralateral target-matching task with smaller VAR JPS errors when listening to sad music as compared to happy or neutral music. (**E**) Participants performed contralateral target-matching tasks with smaller VAR JPS errors when matching with their dominant vs. non-dominant feet. (**F**) Participants who listened to happy music during the contralateral target-matching tasks had lower maximal heart rate when matching with their non-dominant vs. dominant feet. (**G**) Participants performed the ipsilateral target-matching task with smaller ABS JPS errors when listening to sad as compared to neutral music. (**H**) Participants performed the ipsilateral target-matching tasks more accurately when matching with their dominant vs. non-dominant feet. (**I**) Participants had lower HR mean during the ipsilateral target-matching task when matching with their non-dominant vs. dominant feet. The violin plots (transparent color) over the boxplots represent the data distribution. The boxplots show the median, the upper, and lower quartiles, and the min and max values. Red error bars within the boxplots represent the 95% confidence interval (CI) around the mean (red dot). Each data point is an individual token: the horizontal jitter is not meaningful and is only used for visualization purposes. *p < 0.05 for post-hoc independent samples- or paired-samples t-test based on a significant Group or Leg main effect, respectively.

Table 1 summarizes the effects of emotions on JPS errors and HR-related data during the contralateral target-matching task with the dominant and non-dominant foot. There were Group main effects for both ABS (F_3,36_ = 14.057, p < 0.001, η_p_^2^ = 0.539) (Fig. 2A) and VAR (F_3,36_ = 8.164, p < 0.001, η_p_^2^ = 0.405) (Fig. 2D). Post-hoc analysis revealed smaller JPS errors in the sad vs. happy (ABS: p < 0.001, d = 1.6; VAR: p = 0.010, d = 1.2) or neutral (ABS: p < 0.001, d = 1.6; VAR: p < 0.001, d = 1.4) group. Statistical analysis also revealed significant Leg main effects in ABS (F_1,36_ = 35.901, p < 0.001, η_p_^2^ = 0.499) (Fig. 2B) and VAR (F_1,36_ = 9.868, p = 0.003, η_p_^2^ = 0.215) (Fig. 2E) JPS errors with the post-hoc analysis showing smaller ABS (d = 0.8) and VAR (d = 0.3) JPS errors during the dominant vs. non-dominant ankle joint’s target-matching. No main or interaction effects were found in CONST JPS errors (all p > 0.05).

**Table 1:** Effects of emotions on joint position sense errors and heart rate-related data during contralateral target-matching task with the dominant and non-dominant foot.

	JPS errors			HR-related data				
	ABS (°)^†^^,^*	CONST (°)	VAR (°)^†^^,^*	Mean (bpm)	Min (beats)	Max (beats)*	Median (bpm)*	HRV (ms)*
Dominant leg								
Happy	4.7 (1.3)	− 0.3 (3.6)	3.8 (1.5)	69.9 (10.6)	63.9 (10.7)	82.5 (10.7)	70.9 (11.7)	11.48 (3.49)
Neutral	4.5 (1.5)	0.9 (3.2)	4.1 (1.4)	73.8 (12.4)	67.5 (11.6)	81.6 (14.5)	73.7 (12.3)	10.07 (4.54)
Sad	2.4 (0.5)	0.8 (1.0)	2.4 (0.7)	67.7 (12.5)	63.0 (11.8)	74.6 (13.2)	67.4 (12.6)	11.63 (5.14)
No music	3.5 (0.8)	2.1 (2.1)	2.8 (0.6)	66.6 (5.0)	61.9 (4.6)	73.4 (6.5)	66.7 (5.0)	9.45 (3.64)
Non-dominant leg								
Happy	6.4 (2.5)	3.1 (6.0)	3.8 (0.6)	69.4 (12.0)	63.7 (11.1)	76.8 (10.0)	67.7 (11.6)	10.45 (4.76)
Neutral	6.8 (2.4)	3.0 (5.5)	4.4 (1.5)	74.6 (12.0)	67.7 (11.3)	81.5 (14.5)	74.0 (11.8)	11.34 (3.42)
Sad	3.6 (0.8)	1.6 (2.0)	3.0 (0.3)	67.0 (12.8)	62.9 (12.6)	72.8 (13.2)	66.7 (12.9)	9.40 (3.58)
No music	4.4 (1.4)	1.9 (3.3)	3.2 (0.4)	66.6 (5.8)	62.0 (5.3)	73.1 (6.7)	66.2 (5.9)	8.88 (3.75)

Values are mean (SD) of each variable.

*ABS* absolute JPS errors, *CONST* constant JPS errors, *HR* heart rate, *HRV* heart rate variability, *JPS* joint position sense, *VAR* variable JPS errors.

^†^Group main effect.

*Leg main effect.

Regarding the HR-related variables, HR median and max showed Leg main effects (each p ≤ 0.004) and Leg × Group interaction effects (each p ≤ 0.006) with the post-hoc analysis revealing lower median (p = 0.024, d = 0.01, Fig. 2C) and maximal heart rate (p = 0.012, d = 0.6, Fig. 2F) in the non-dominant vs. dominant feet of participants in the happy group.

No correlation was found between any type of JPS errors and HR-related data (all p > 0.05).

### Ipsilateral matching task

Table 2 shows the effects of emotions on JPS errors and HR-related data during the ipsilateral target-matching task with the dominant and non-dominant foot. Significant Group main effect was found in ABS JPS errors (F_3,36_ = 4.229, p = 0.012, η_p_^2^ = 0.261). Post-hoc analyses revealed smaller JPS errors in the sad as compared to neutral (ABS: p = 0.007, d = 1.2, Fig. 2G) group. There was also a Leg main effect in ABS JPS errors (F_1,36_ = 18.593, p < 0.001, η_p_^2^ = 0.341) with the post-hoc analysis showing less JPS errors during dominant vs. non-dominant ankle joint’s target-matching (d = 0.4) (Fig. 2H). No main or interaction effects were found in CONST or VAR JPS errors (all p > 0.05).

**Table 2:** Effects of emotions on joint position sense errors and heart rate-related data during ipsilateral target-matching task with the dominant and non-dominant foot.

	JPS errors			HR-related data				
	ABS (°)^†^^,^*	CONST (°)	VAR (°)	Mean (bpm)*	Min (beats)	Max (beats)*	Median (bpm)	HRV (ms)^†^
Dominant leg								
Happy	2.6 (0.8)	0.2 (1.6)	2.9 (0.8)	73.9 (11.6)	65.6 (11.4)	87.5 (11.7)	72.9 (12.2)	10.86 (2.99)
Neutral	3.3 (1.2)	1.0 (2.5)	3.3 (0.7)	75.0 (11.5)	67.4 (9.6)	84.9 (12.9)	75.0 (11.8)	9.88 (4.60)
Sad	2.2 (0.5)	0.4 (0.9)	2.5 (0.6)	68.1 (12.5)	63.1 (11.9)	76.6 (11.9)	67.9 (12.9)	11.93 (5.26)
No music	2.6 (0.5)	1.0 (1.3)	2.7 (0.5)	67.6 (5.2)	62.1 (4.6)	76.6 (4.3)	67.3 (5.2)	9.21 (3.60)
Non-dominant leg								
Happy	3.0 (0.9)	0.1 (2.0)	3.2 (0.9)	71.7 (14.3)	64.5 (12.3)	81.8 (15.5)	71.5 (14.5)	9.37 (3.88)
Neutral	4.0 (1.7)	2.0 (2.8)	3.4 (0.8)	74.0 (12.1)	67.1 (11.0)	83.8 (13.5)	74.1 (11.7)	10.45 (2.95)
Sad	2.5 (0.7)	0.5 (1.1)	2.7 (0.9)	67.0 (13.0)	61.8 (11.3)	73.4 (13.6)	66.9 (13.1)	9.54 (3.87)
No music	3.1 (0.7)	0.9 (1.6)	3.1 (0.5)	67.1 (5.3)	62.1 (5.3)	74.7 (5.8)	67.0 (5.3)	8.43 (3.48)

Values are mean (SD) of each variable.

*ABS* absolute JPS errors, *CONST* constant JPS errors, *HR* heart rate, *HRV* heart rate variability, *JPS* joint position sense, *VAR* variable JPS errors.

^†^Group main effect.

*Leg main effect.

Statistical analyses revealed Leg main effects in HR mean (F_1,36_ = 7.581, p = 0.009, η_p_^2^ = 0.174) and max (F_1,36_ = 9.663, p = 0.004, η_p_^2^ = 0.212) with the post-hoc analyses showing lower HR-related values when matching with the non-dominant vs. dominant feet (HR mean: d = 0.1, HR max: d = 0.244) (Fig. 2I).

In line with our findings on contralateral matching tasks, no significant correlation was found between HR-related data and JPS errors.

## Discussion

In the present pilot study, we examined if HR-related physiological changes induced by music-elicited emotions would underlie alterations in healthy young adults’ ankle joint target-matching strategy quantified by JPS. Although classical music-elicited emotions affected target-matching errors and HR-related data, these two outcomes were uncorrelated. In line with our hypothesis, participants who listened to classical music pieces that elicited happy emotions performed the target-matching task less accurately as compared to those who listened to sad music. Lastly, participants performed the target-matching tasks more accurately with their dominant vs. non-dominant limb, regardless of emotional state and the task. Overall, our results suggest that emotions have the potential to affect target-matching strategy, however, contrary to our hypothesis, HR-related physiologic changes did not underlie the alteration of ankle joint target-matching strategy in response to classical music-elicited emotions.

Our results are partly in line with previous studies, i.e., participants who listened to sad music during the contralateral target-matching task produced smaller ABS JPS (2.4 ± 0.5°) errors as compared to those who listened to happy (4.7 ± 1.3°) or neutral (4.5 ± 1.5°) music. Sad emotions can increase muscle spindle dynamic response, which in turn allows us to prime movements so that proprioceptive acuity sharpens^3^. Indeed, a previous study showed that emotional state modulated kinesthetic acuity: it was higher in response to listening to sad vs. no music or neutral conditions^6^. However, in the present study, none of the groups who listened to classical music differed from the control (no music) condition. Although participants in the previous and the present studies listened to nothing during the experiment through noise-cancelling headphones, it is possible that the inconsistent results are due to the different experimental modalities. Still, feeling sadness seems to strongly facilitate perceptual and neural responses in both studies. One possible explanation for this is that sad feeling may be more important for survival^39^ and prime the body for a context-appropriate behavioural response, e.g., withdrawal and avoidance. Another explanation for the differences in ABS JPS errors between the sad and happy groups is that the happy music may have evoked a desire to move, however, the participants were required to remain relaxed for the experiment, which may have contributed to the larger target-matching errors. Finally, attention appears particularly important in our research. Previous studies investigated different types of attentional processes and their interactions with emotion. Emotion-attention interactions involve interplays between affective and executive brain systems^40^. More than two decades ago it was shown that emotional information modulates two anatomically and functionally distinct attentional systems in the brain^41^. The interaction between emotion and attention is complex and intertwined, can involve both top-down/goal-driven allocation of attention and bottom-up/stimulus-driven attentional processes, and is linked to early stages of perceptual processing. It is, therefore, possible that listening to sad classical music pieces appeared to be most efficient in facilitating perceptual responses due to more urgent arousal or attentional levels^39^, while the happy music may draw the participant’s attention away from the JPS task.

The analysis of the direction of error (CONST) did not show statistically significant main or interaction effects most probably due to the relatively small average target-matching errors with large inter-subject variability. Although participants tended to overestimate the target positions in each matching task, leg and group, participants in the happy group performed the target-matching task with underestimation (− 0.3 ± 3.6°). Nevertheless, considering the lack of statistically significant results, its practical significance is also questionable. Overall, it seems that in our study, emotions did not affect participants’ motor control strategy in terms of target under- or overestimation suggesting that participants in each group probably used more than one motor control strategy during contralateral and ipsilateral JPS tasks, which resulted in their more random performance.

In addition, variability in human voluntary movements is essential for flexibility and stability^42^. Such observations make assessing variable JPS errors relevant. These types of errors compared with absolute errors provide different information on the integrity of the sensorimotor system^31,32^. In the present study, participants who listened to classical music pieces that were deemed to evoke sad feelings had smaller VAR JPS errors (3.6 ± 0.8°) as compared to those who listened to happy (6.4 ± 2.5°) or neutral (6.8 ± 2.4°) music. Considering that the neuromuscular system gets noisier and less adaptable^43^ when variability is increased above an optimum level, our results suggest that feeling sadness had favorable effects on the variability of contralateral target-matching errors compared to the other two feelings. Interestingly, the more constant target-matching strategy, indicated by VAR JPS errors, did not appear during the ipsilateral matching condition, i.e., no differences were found between the four groups (Table 2). Given that the contralateral tasks involve interhemispheric transfer of information, while ipsilateral tasks are memory-based^16,17^, it is possible that the contralateral vs. ipsilateral target-matching strategy is related to different activation patterns^17^ or interhemispheric brain area coherence, especially when the target-matching task is done in different emotional states.

As a second aim, we also determined whether participants would perform the target-matching tasks more accurately with their non-dominant vs. dominant limb during both contralateral and ipsilateral conditions, independent of their emotional state. We addressed this hypothesis based on previous behavioural^23,24^ and neuroimaging^17^ data indicating right hemisphere dominance during proprioceptive position sense. Specifically, extensive literature suggests that right-side dominant participants perform lower^24^ and upper limb joints [thumb^44,45^, elbow^23,46,47^, multiple joints (ankle, knee, shoulder, finger)^48^] proprioceptive target-matching tasks more accurately when using their non-dominant left limb as compared with left-side dominant participants performing the same task with their non-dominant right limb joints. Such lateralization of proprioception-related functional activity is supported by neuroimaging data showing a right hemisphere dominance in limb movement perception^17,49–51^. However, our results indicate that participants in both target-matching conditions performed the tasks more accurately with their dominant vs. non-dominant feet. We cannot conclude that target-matching asymmetry would be altered by emotions considering that participants who did not listen to any kind of music during the experiment also produced smaller JPS errors when they were matching with their dominant foot. One possible reason for these unexpected findings could be the use of ankle joints in the present study. Most of the previous studies that compared the dominant and non-dominant limb’s proprioceptive acuity used the knee joint to determine JPS errors^24,52–54^. To the best of our knowledge, studies that aimed to determine the JPS of both the dominant and non-dominant ankles are limited. A previous study^55^ that aimed to establish the use of ankle tape on ankle JPS did not report any differences between the dominant and non-dominant limb’s target matching errors. Overall, our results may suggest that target-matching asymmetry might be different in the ankle joint as compared to any other upper or lower-limb joints.

In addition to differences in JPS errors, some classical music-elicited emotional states seem to affect HR-related data. Specifically, during the contralateral matching task, participants who listened to happy music had lower median and maximal HR when matching with their non-dominant vs. dominant feet. On the other hand, previous studies^6,19,20^ reported larger HRV for participants who listened to happy music as compared to those who listened to sad music. In the present study, we used the LF/HF ratio as a measure of HRV, which was reported to be higher in individuals with posttraumatic stress disorder as compared to healthy controls^56^, therefore, it could be used as an indicator of stress. Although our results indicated no group differences or association between HR-related data and any type of JPS errors, the elevated values in each matching task and group indicated SNS domination suggesting that each participant experienced higher stress levels, regardless of emotion group. Nevertheless, the data suggest that HR-related physiologic changes do not underlie the alteration of ankle joint target-matching strategy, irrespective of the emotion. It seems that while emotions can modulate muscle afferent discharge^4,5^ and these afferents also control JPS^6^, the co-occurrence of afferent behavior is incidental and physiologically unrelated.

### Limitations and future perspectives

The main limitation of the study is the use of a parallel study design. It would be important to see whether classical music pieces that were considered to elicit different emotions would affect the proprioceptive acuity of the same sample. However, given the relatively long experiment used in the present study, we believe that setting up a parallel study design versus a crossover design was more feasible to address the hypotheses. Nevertheless, future studies should clarify the effect of emotion on ankle joint proprioception using a simplified study design. In line with this, some HR-related variables violated the assumption of normality (Shapiro–Wilk test) even after log transformation (Supplementary Table 2) most probably due to the relatively low sample size in each group. However, considering that our results indicate that HR-related physiologic changes do not underlie the alteration of ankle joint target-matching strategy in response to classical music-elicited emotions, this did not affect the main outcome of the study.

Another limitation of the study is that the present study did not take participants’ range of motion (ROM) into account. Therefore, considering that target matching positions were the same for each participant, participants with different ROM would experience different musculoskeletal tension in the same position, which potentially affected JPS. Future studies should clarify the significance of maximal ROM on target-matching accuracy. Moreover, supplementing the behavioral data with electroencephalography (EEG) recordings could identify whether participants' target-matching strategies are related to coherence or activation patterns of brain areas when emotions are altered.

Our unexpected results regarding target-match asymmetry should be further investigated in future studies to determine whether JPS would consistently be more accurate if participants performed the task with their dominant vs. non-dominant ankle joint. Although we have no reliability data for the custom-made foot platform, the careful randomization of the conditions and target angles (Supplementary Data 1–4) minimized any bias due to learning. Therefore, this factor is less likely to be the reason for the unexpected results.

In the present study, we used JPS as a valid assessment of proprioception considering that previous studies targeted the effects of emotion only on muscle spindles’ dynamic response^3–5^. However, because emotions may affect other proprioceptors, i.e., Golgi tendon organs, tactile receptors and other interoceptors differently, future studies should improve the experimental paradigms^57^ by quantifying movement extent, trajectory, velocity and the sense of force, and muscle tension. Such measurements would allow researchers to determine the effects of emotion on all aspects of proprioception.

Finally, although the differences in JPS errors were significant between the groups, these differences were quite small. Whether such minimal detectable differences have any physiological or functional importance is unclear. Future studies should determine if classical music-induced emotions would induce larger differences in JPS errors after an ankle injury, i.e., in recurrently sprained ankle or in functional ankle instability.

## Conclusions

Overall, our results indicate that emotions have the potential to affect target-matching strategy, however, contrary to our hypothesis, (1) participants performed both contralateral and ipsilateral target-matching tasks with smaller JPS errors when matching with their dominant vs. non-dominant foot and (2) HR-related physiologic changes do not underlie the alteration of ankle joint target-matching strategy in response to classical music-elicited emotions. Future studies should further clarify target-matching asymmetry in the ankle joint and whether emotions would induce different brain area coherence or activation patterns during such target-matching tasks to identify the potential underlying mechanisms of emotion-affected proprioception.

### Supplementary Information

Supplementary Information.

## Data Availability

The datasets used and/or analyzed during the current study are presented within the paper and its Supporting Information files. Additional raw data are also available from the corresponding author upon reasonable request.

## Abbreviations

ABS: Absolute target-matching errors
BAS: The foot platform’s baseline resting position
CON: Control group receiving no music during the target-matching tasks
CONST: Constant target-matching errors
DF: Dorsiflexion
HR: Heart rate
HRV: Heart rate variability
JPS: Joint position sense
PF: Plantarflexion
rANOVA: Repeated measures variance of analyses
REF: The foot platform’s reference position with the foot oriented at 90°
VAR: Variable target-matching errors
VAS: Visual analogue scale
