# Supplementary material for: Heart rate-related physiological changes induced by classical music-elicited emotions do not underlie alterations in healthy adults’ ankle joint target-matching strategy
Source: Sci Rep. 2024 Jul 17;14:16482. doi: 10.1038/s41598-024-67467-y (PMC11252265; doi:10.1038/s41598-024-67467-y)
Supplement: Supplementary file 1 — Supplementary Information. [file 41598_2024_67467_MOESM1_ESM.docx]

*Supplementary material*

**Title:** Heart rate-related physiological changes induced by classical music-elicited emotions do not underlie alterations in healthy adults’ ankle joint target-matching strategy

**Journal name:** nature Scientific Reports

**Authors:** Keqing Yuan^1^, Takeshi Okuyama^2^, Tibor Hortobágyi^3, 4, 5^, Ryoichi Nagatomi^1, 6^, János Négyesi^3, 7, 8, *^

**Affiliations:** ^1^Department of Medicine and Science in Sports and Exercise, Tohoku University Graduate School of Medicine, Sendai, Japan, ^2^Department of Robotics, Tohoku University Graduate School of Engineering, Sendai, Japan; ^3^Department of Kinesiology, Hungarian University of Sports Science, Budapest, Hungary; ^4^Department of Human Movement Sciences, Center for Human Movement Sciences, University Medical Center Groningen, University of Groningen, Groningen, The Netherlands; ^5^Institute of Sport Sciences and Physical Education, University of Pécs, Pecs, Hungary; ^6^Department of Biomedical Engineering for Health Maintenance and Promotion, Graduate School of Biomedical Engineering, Tohoku University, Sendai, Miyagi, Japan; ^7^Neurocognitive Research Center, Nyírő Gyula National Institute of Psychiatry, and Addictology, Budapest, Hungary; ^8^CRU Hungary Kft., Budapest, Hungary

**Corresponding author:**

János Négyesi

e-mail: negyesi.janos@tf.hu

| **Supplementary Table 1:** The set of classical music pieces used in the study to induce different emotional states | | | | |
| --- | --- | --- | --- | --- |
| **Composer** |  | **Music piece** |  | **Valence** |
| Mozart |  | Piano Concerto No. 23, Allegro assai |  | Happy |
| Bizet |  | Carmen Suite No. 1, Les Toreadors |  | Happy |
| Saint-Saeëns |  | Carnival of the Animals No. 10, Aviary |  | Happy |
| Vivaldi |  | Four seasons, Spring, Allegro |  | Happy |
| Strauss |  | Radetzky marsch, Op. 228 |  | Happy |
| Beethoven |  | Symphony No. 3 in E flat major ('Eroica'), Op. 55- Scherzo, Allegro vivace |  | Happy |
| Mozart |  | Eine kleine Nachtmusik, K. 525 |  | Happy |
| Vivaldi |  | Four seasons, Autumn, Allegro |  | Happy |
| Saint-Saeëns |  | Carnival of the Animals No. 14, Finale |  | Happy |
| Beethoven |  | Symphony No.6 In F Major, Op.68 - III. Allegro |  | Happy |
|  |  |  |  |  |
| Handel |  | Water music suite No. 1 Minuet (mvt 6) |  | Neutral |
| Handel |  | Water music suite No. 2 passepied (mvt 2) |  | Neutral |
| Holst |  | The Planets, Venus |  | Neutral |
| Beethoven |  | Piano Sonata No. 14, Moonlight Sonata |  | Neutral |
| Mussorgsky |  | Pictures at an exposition No. 1 |  | Neutral |
|  |  |  |  |  |
| Bruch |  | Kol Nidrei, Op. 47 |  | Sad |
| Chopin |  | Nocturnes, Op. 27 No. 1 |  | Sad |
| Rodrigo |  | Concerto De Aranjuez, Adagio (mvt 2) |  | Sad |
| Malher |  | Symphony No. 5, Adagietto |  | Sad |
| Chopin |  | Nocturnes, Op. 48 No. 1 |  | Sad |
| Barber |  | Adagio for Strings |  | Sad |
| Albinoni |  | Adagio |  | Sad |
| Schubert |  | String Quartet No. 14 |  | Sad |
| Rachmaninov |  | Piano Concerto No. 2, Adagio |  | Sad |
| Ravel |  | Piano Concerto in Sol (mvt 2) |  | Sad |
| Mozart |  | Piano Concerto No. 23, Adagio |  | Sad |
| Saint-Saeëns |  | Carnival of the Animals No. 13, The swan |  | Sad |
| Grieg |  | Peer Gynt: Solveig’s song, Op. 55 No. 2 |  | Sad |
| Adopted from Ackerley et al. 2017^1^  The pre-determined emotional valence of each music piece was determined in previous studies^2-7^. | | | | |

**References**

1 Ackerley, R., Aimonetti, J. M. & Ribot-Ciscar, E. Emotions alter muscle proprioceptive coding of movements in humans. Sci Rep-Uk 7, (2017).

2 Peretz, I., Gagnon, L. & Bouchard, B. Music and emotion: perceptual determinants, immediacy, and isolation after brain damage. Cognition 68, 111-141 (1998).

3 Mitterschiffthaler, M. T., Fu, C. H., Dalton, J. A., Andrew, C. M. & Williams, S. C. A functional MRI study of happy and sad affective states induced by classical music. Hum Brain Mapp 28, 1150-1162 (2007).

4 Baumgartner, T., Esslen, M. & Jäncke, L. From emotion perception to emotion experience: emotions evoked by pictures and classical music. Int J Psychophysiol 60, 34-43 (2006).

5 Baumgartner, T., Lutz, K., Schmidt, C. F. & Jäncke, L. The emotional power of music: how music enhances the feeling of affective pictures. Brain Res 1075, 151-164 (2006).

6 Baumgartner, T., Willi, M. & Jäncke, L. Modulation of corticospinal activity by strong emotions evoked by pictures and classical music: a transcranial magnetic stimulation study. Neuroreport 18, 261-265 (2007).

7 Krumhansl, C. L. An exploratory study of musical emotions and psychophysiology. Can J Exp Psychol 51, 336-353 (1997).

| **Supplementary Table 2:** List of variables that violated the assumption of normality even after log transformation | | | | | | |
| --- | --- | --- | --- | --- | --- | --- |
|  |  | Variables not normally distributed |  | Variables still not normally distributed after log transformation |  | Shapiro-Wilk Sig. |
| *Contralateral* |  |  |  |  |  |  |
| JPS_ABS |  | - |  | - |  | - |
| JPS_CONST |  | - |  | - |  | - |
| JPS_VAR* |  | N_ND |  | H_ND |  | 0.029 |
|  |  |  |  |  |  |  |
| HR_Mean* |  | N_D |  | N_D |  | 0.017 |
|  |  | N_ND |  | N_ND |  | 0.015 |
| HR_Median* |  | N_D |  | N_D |  | 0.007 |
|  |  | N_ND |  | N_ND |  | 0.008 |
| HR_Min* |  | N_D |  | N_D |  | 0.014 |
|  |  | N_ND |  | N_ND |  | 0.027 |
| HR_HRV |  | H_ND |  | - |  | 0.644 |
| *Ipsilateral* |  |  |  |  |  |  |
| JPS_ABS |  | N_ND |  | - |  | 0.084 |
| JPS_CONST |  | - |  | - |  | - |
| JPS_VAR |  | - |  | - |  | - |
|  |  |  |  |  |  |  |
| HR_Mean* |  | N_D |  | N_D |  | 0.005 |
|  |  | N_ND |  | N_ND |  | 0.002 |
| HR_Median* |  | N_D |  | N_D |  | 0.005 |
|  |  | N_ND |  | N_ND |  | 0.005 |
| HR_Min* |  | N_ND |  | N_ND |  | 0.012 |
| HR_Max* |  | N_ND |  | N_ND |  | 0.028 |
| HR_HRV* |  | N_D |  | H_D |  | 0.037 |
| Abbreviations: ABS: absolute target-matching error; CONST: constant target-matching error; H: happy group; HR: heart rate; HRV: heart rate variability; JPS: joint position sense; N: neutral group; VAR: variable target-matching error  *violated the assumption of normality even after log transformation | | | | | | |

**Supplementary Data 1:** Random number generation for dominant foot ipsilateral JPS tasks.

**Supplementary Data 2:** Random number generation for non-dominant foot ipsilateral JPS tasks.

**Supplementary Data 3:** Random number generation for dominant foot contralateral JPS tasks.

**Supplementary Data 4:** Random number generation for non-dominant foot contralateral JPS tasks.
